# Supplementary material for: Downregulation of Glycine N-Acyltransferase in Kidney Renal Clear Cell Carcinoma: A Bioinformatic-Based Screening
Source: Diagnostics (Basel). 2023 Nov 22;13(23):3505. doi: 10.3390/diagnostics13233505 (PMC10706668; doi:10.3390/diagnostics13233505)
Supplement: Supplementary file 1 [file diagnostics-13-03505-s001.zip › diagnostics-2671654-supplementary figures.pdf]

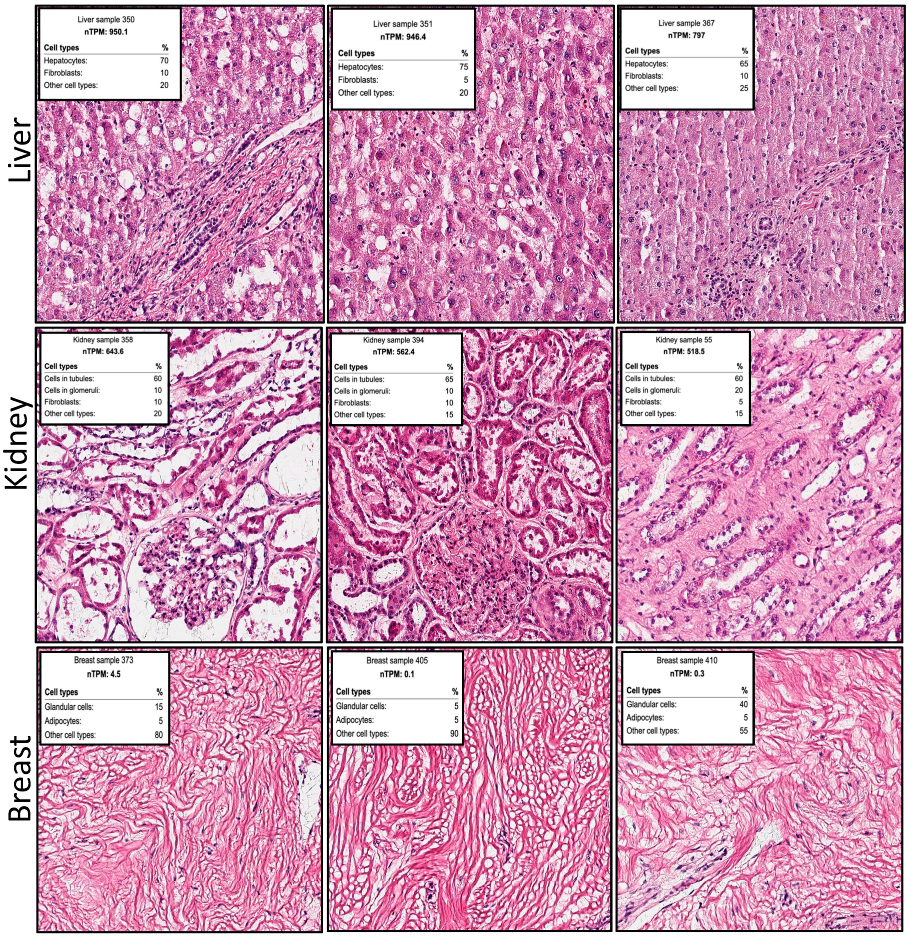

Figure S1. Hematoxylin and eosin staining of normal liver, kidney, and breast tissue used for transcriptomic profiling analysis. Images credit: HPA.

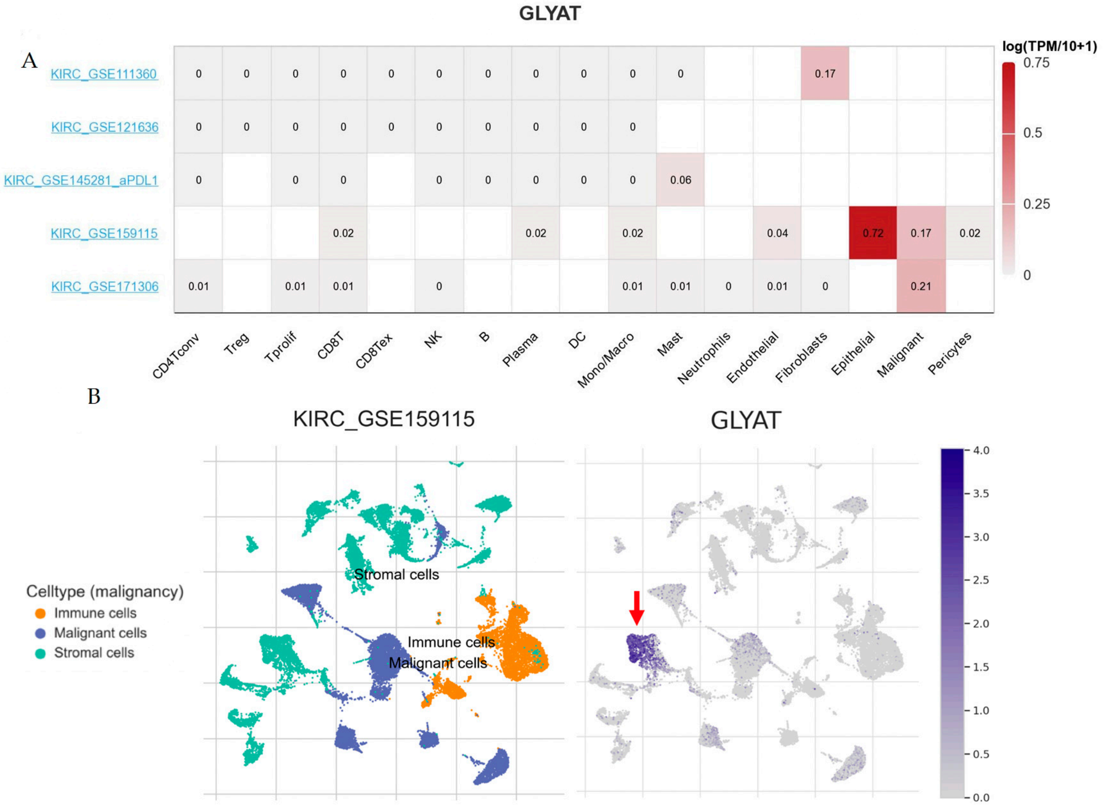

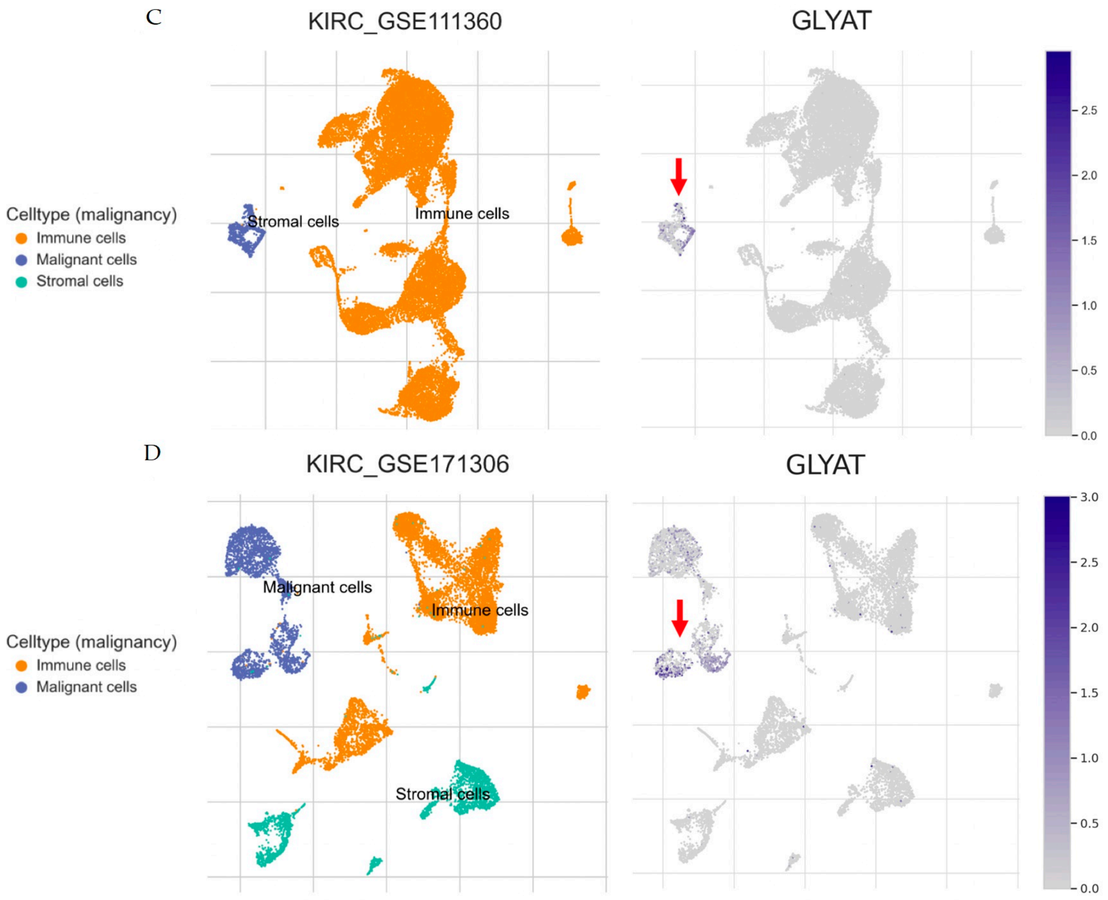

Figure S2. (**A**) Average gene expression of GLYAT on tumor microenvironment cells in KIRC. Plot represents scRNA-seq data obtained from the TISCH2 Web tool. Uniform Manifold Approximation and Projection (UMAP) showing GLYAT expression in GSE159115 (**B**), GSE111360 (**C**), and GSE171306 (**D**) datasets derived from primary tumors of KIRC. *** $p < 0.001$, and **** $p < 0.0001$.
